# No protein intake compensation for insufficient indispensable amino acid intake with a low-protein diet for 12 days

**DOI:** 10.1186/1743-7075-11-38

**Published:** 2014-08-20

**Authors:** Eveline A Martens, Sze-Yen Tan, Richard D Mattes, Margriet S Westerterp-Plantenga

**Affiliations:** 1 Department of Human Biology, Nutrition and Toxicology Research Institute Maastricht (NUTRIM), Maastricht University, PO Box 616, 6200, MD Maastricht, The Netherlands; 2 Department of Nutrition Science, Purdue University, West Lafayette, IN 47905, USA

**Keywords:** Protein intake, Nitrogen balance, Indispensable amino acids, DIAAS, Protein source

## Abstract

**Background:**

Protein quality evaluation aims to determine the capacity of food sources and diets to meet protein and indispensable amino acid (IAA) requirements. This study determined whether nitrogen balance was affected and whether dietary IAA were adequately obtained from the ad libitum consumption of diets at three levels of protein from different primary sources for 12 days.

**Methods:**

Two 12-day randomized crossover design trials were conducted in healthy subjects [*n* = 70/67 (M/F); age: 19-70 y; BMI: 18.2-38.7 kg/m^2^]. The relative dietary protein content was lower than [5% of energy (En%)], similar to (15En%), and higher than (30En%) customary diets. These diets had a limited variety of protein sources, containing wheat protein as a single protein source (5En%-protein diet) or 5En% from wheat protein with 10En% (15En%-protein diets) or 25En% (30En%-protein diets) added from whey with α-lactalbumin, soy or beef protein.

**Results:**

There was a dose-dependent increase in nitrogen excretion with increasing dietary protein content, irrespective of the protein sources (*P* = 0.001). Nitrogen balance was maintained on the 5En%-protein diet, and was positive on the 15En%- and 30En%-protein diets (*P* < 0.001) over 12 days. Protein intake from the 5En%-protein diet did not reach the amount necessary to meet the calculated minimal IAA requirements, but IAA were sufficiently obtained from the 15En%- and 30En%-protein diets. In the 15En%- and 30En%-protein conditions, a higher protein intake from the soy-containing diets than from the whey with α-lactalbumin or beef containing diets was needed to meet the minimal IAA requirements.

**Conclusion:**

Protein intake did not compensate for an insufficient indispensable amino acid intake with a low-protein diet for 12 days.

**Trial registration:**

These trials were registered at clinicaltrials.gov as NCT01320189 and NCT01646749.

## Background

Meeting metabolic needs for amino acids is crucial to ensure health [1]. Amino acids provide nitrogen and carbon skeletons for tissue protein synthesis and for the production of nitrogenous compounds involved in a range of bodily functions [2]. Many types of amino acids are needed physiologically but some cannot be synthesized in the body and must therefore be obtained through the diet; these amino acids are known as the dietary indispensable amino acids (IAA). Protein quality evaluation aims to determine the capacity of food sources and diets to meet protein and IAA requirements. Currently, nitrogen balance studies are primarily applied to define protein requirements as the degree to which protein intake compensates for obligatory losses of nitrogen from several pathways including amino acid breakdown [1,3]. However, nitrogen balance alone may be insufficient and other factors should be considered when determining protein requirement. For example, protein sources may influence the efficiency of protein utilization, and diets containing protein of lower quality are associated with increased losses of nitrogen [2,4,5]. Furthermore, the observation of a non-linear relationship between protein intake and nitrogen balance indicates the existence of metabolic adjustments to changes in protein intake [2,6]. The overall requirements of amino acids should consider adaptive components, such as net protein deposition and oxidative losses [3].

Due to a large inter-individual variability, even during energy balance and after metabolic adaptation, nitrogen balance studies are not sufficiently accurate to determine whether dietary protein intake meets IAA requirements [1,3,7]. In the context of protein-diets, the digestible IAA score (DIAAS) enables the determination whether dietary protein intake is sufficient for the metabolic needs for the individual IAA. The DIAAS permits the definition of protein quality by determination of the extent to which dietary IAA are provided in proportion to the requirements [1].

Recently, it has been proposed that humans either consume relatively more energy from a low-protein diet [8], or relatively less from a high-protein diet [9,10]. In these studies, complete protein leveraging was absent [8-10]. It is unclear whether protein intake compensates for the protein-proportions to maintain nitrogen balance or to meet a sufficient amount of IAA. Compensatory intake of protein for obligatory loss of nitrogen is essential for normal growth and development in organisms [1,3]. The proposed mechanism to prevent negative nitrogen balance may be related to an observed signaling pathway that detects amino acid depletion [11-19]. This study determined whether nitrogen balance was affected and whether dietary IAA were adequately obtained from the ad libitum consumption of diets at three levels of protein from different primary sources for 12 days. Data from two dietary intervention trials conducted over 12 consecutive days were combined and analyzed [9,10]. The relative dietary protein content was lower than [5% of energy (En%)], similar to (15En%), and higher than (30En%) customary diets. These diets had a limited variety of protein sources, containing wheat protein as single protein source (5En%-protein diet) or 5En% from wheat protein with 10En% (15En%-protein diets) or 25En% (30En%-protein diets) added from whey with α-lactalbumin, soy or beef protein.

## Methods

Results from a trial on whey protein with α-lactalbumin (*n* = 39) and soy protein (*n* = 40) at Maastricht University [9], and a two-center trial on beef protein (*n* = 58) at Maastricht University and at Purdue University [10] were combined for analysis. These studies were conducted according to the guidelines laid down in the Declaration of Helsinki and all procedures involving human subjects were approved by the Medical Ethical Committees of Maastricht University and Purdue University. Written informed consent was obtained from all subjects. The study on whey with α-lactalbumin and soy protein was registered as NCT01320189 and that on beef protein as NCT01646749 at clinicaltrials.gov. Data on energy and protein intake of the separate studies has been previously published [9,10]. The present study encompasses the analyses on the nitrogen balances and on the DIAAS.

### Study subjects

One hundred and eighty-six subjects were recruited, of whom 28 dropped out due to lack of time. Four subjects were excluded from the data analysis because of non-compliance, as shown by urinary nitrogen excretion. Overall, 137 subjects (70 men and 67 women) were included in the final data analysis. BMI ranged from 18.2-38.7 kg/m^2^ and age from 19 to 70 y [9,10]. Subjects underwent screening that included anthropometric measurements and the completion of questionnaires eliciting information about health, smoking behavior, use of medications, alcohol consumption, physical activity, eating behavior and liking of the study meals. Subjects were non-smoking, not using more than moderate amounts of alcohol (>10 drinks/wk), were weight stable (body weight change < 3 kg during the prior 6 mo and had no planned weight change during the study period), were not using medication or supplements except for oral contraceptives in women, and rated the taste of the meals as acceptable [Visual analog scale (VAS) score for liking: ≥ 50 mm] [9,10]. Individual daily energy requirements were calculated as the basal metabolic rate calculated with the formula of Harris and Benedict [20], times the physical activity level based on the Baecke Activity Questionnaire or on the two validated questions physical activity at work and at leisure time [21,22].

### Study design

In the two single-blind, randomized crossover design trials, the relative protein content included 5En% from wheat protein (5En%-protein diet), and 5En% from wheat protein with 10En% (15En%-protein diets) or 25En% (30En%-protein diets) from whey with α-lactalbumin (Hiprotal Whey Protein Alpha; DOMO, FrieslandCampina), soy protein (SUPRO Soy Protein Isolate; Solae LLC) or beef protein. Subjects were randomly assigned to diets containing whey protein with α-lactalbumin or the soy protein in trial one and all consumed beef protein in the second trial. The order of the three treatment arms for protein energy content was random for all subjects. Each dietary intervention lasted for a period of 12 consecutive days, in which subjects visited one of the universities for ad libitum consumption of breakfasts, lunches and dinners [9,10]. Prior work indicated that this period of intervention is long enough to reliably measure possible effects on energy and macronutrient intake [23]. The wash-out period between the test sessions was ~ 6 wk to minimize carry-over effects and to take menstrual cycle phase effects into account in women on energy intake [24,25].

### Diet composition

Each protein-diet consisted of 5En% from wheat flour protein. The 5En%-protein condition contained wheat flour as a single protein source. In the 15%-protein conditions, the protein content was comprised of 33% (5En%) of wheat flour protein and of 67% (10En%) of either whey plus α-lactalbumin, soy or beef protein. Dairy protein was comprised of 70% of whey protein and of 30% of α-lactalbumin. Protein content of the 30En%-protein conditions comprised of 17% (5En%) of wheat flour protein and of 83% (25En%) of either beef, whey plus α-lactalbumin or soy protein. Again, dairy protein content was comprised of 70% of whey protein and of 30% of α-lactalbumin. The fat content between the meals and between the conditions was maintained at a constant proportion (35 En%) to prevent possible effects of energy density and palatability on energy intake [26-28]. The resulting diet compositions were 5/60/35 En% from protein/carbohydrate/fat (low-protein), 15/50/35 En% (medium-protein), and 30/35/35 En% (high-protein). Moreover, the fiber content was comparable between the conditions.

### Biomarker of protein intake and nitrogen balance

Nitrogen excretion, measured from 24-h urine collections at baseline (day 0) and at days 5 and 11, was used as a crude estimate of protein intake. Urine was collected in 2-L urine bottles containing 10 mL of diluted hydrochloric acid (4 mmol/L) to prevent nitrogen loss through evaporation. Collection started after the first voiding in the morning on the collection days at 8.00 h, and lasted through the first voiding on the next day at 8.00 h. The total volume of the 24-h urine was recorded. Urine was gently mixed, and samples were taken and frozen at -20°C until analysis. Nitrogen concentrations were measured with an elemental analyser (CHN-O-Rapid, Heraeus, Hanau, Germany, in the Netherlands, and Integra COBAS 400 plus, Roche Diagnostics GmbH, Rotkreuz, Switzerland, in the USA). Total nitrogen output was calculated as 24-h urinary nitrogen plus 10% to account for normal losses via feces and other losses [9,10]. Nitrogen balance was calculated as the difference between nitrogen excretion and nitrogen intake.

### Protein intake

Each meal was weighed to the nearest gram before it was provided to the subjects. Leftovers were reweighed to permit calculation of protein intake for each subject. Weights of provided snack items were also recorded, and the portions returned uneaten were weighed to determine the protein intake from the snacks per subject. Mean protein intake was calculated as the sum of protein intake from meals and from snacks [9,10].

### DIAAS

The IAA compositions of wheat protein and that of beef protein in the provided diets were obtained from the United States Department of Agricultural data base [29] and from a FAO publication [30] (Additional file 1). Amino acid analyses of specific food products provided the dietary IAA compositions of whey, α-lactalbumin and soy protein (Additional file 1) [31]. The IAA content of the protein-diets was calculated as the sum of IAA of the separate proteins in the diets. Subsequently, the IAA content of the protein-diets was corrected for digestibility (Additional file 2) [1]. Since fecal protein represents largely bacterial protein, determination of undigested protein at the end of the ileum is a more reliable measure for the digestibility of dietary protein [32-34]. Furthermore, the predicted ileal amino acid content needs to be corrected for ileal endogenous amino acids that are voided in the digestive tract. When the coefficients of amino acid digestibility are corrected for the endogenous amino acids, the resultant digestibility coefficients are considered as ‘true’ coefficients [1,32,33]. Differences have been observed in the ileal digestibility of different proteins, as well as between different amino acids within each type of protein [1,32,35]. Therefore, in this study, the digestible IAA content was calculated for each individual IAA within each of the protein sources (wheat, whey with α-lactalbumin, soy, and beef) as the dietary IAA content (mg/g protein) multiplied by the corresponding IAA true ileal digestibility coefficient. True ileal digestibility coefficients were obtained from a compound table representing observations in humans, pigs and rats from single studies or as means across studies [33]. In general, digestibility coefficients for humans are used. When human data were not available, predictions for humans based on data obtained from growing pigs or rats were used, and then followed by digestibility coefficients for growing pigs or rats. For wheat flour protein, a predicted value obtained from pig true ileal amino acid digestibility was used, based on the equations of Deglaire and Moughan and from the endogenous amino acid losses determined using the protein-free diet method [32-34]. A determined value in humans was available for the whey with α-lactalbumin protein concentrate, whereby endogenous amino acid losses were determined using the protein-free diet method [32-34,36]. Also for soy protein isolate, amino acid digestibility coefficients corrected for endogenous amino acid losses were determined using the protein-free diet method. These were available from a human study by Moughan et al. [32-34,36]. Since specific studies on beef protein are scarce, the amino acid digestibility coefficients of meat protein hydrolysate determined in rats with endogenous amino acid losses determined using the enzyme hydrolyzed casein/ultrafiltration method, were used as representatives [33].

The minimal IAA requirements are the minimal intakes of IAA to avoid deficiencies [37,38]. These represent the profile of IAA intake to fulfill the obligatory role of maintenance. The DIAAS for the minimal IAA intakes were calculated for each IAA per protein-diet with the following formula (Additional file 3):

$$\begin{array}{c} \text{DIAAS}\;\left(\%\right)\;=\;100\;\times [(\text{mg}\;\text{of}\;\text{the}\;\text{digestible}\;\text{dietary}\\ \;\text{IAA}\;\text{in}\;1\;g\;\text{of}\;\text{the}\;\text{dietary}\;\text{protein}/\\ \;(\text{mg}\;\text{of}\;\text{the}\;\text{same}\;\text{dietary}\;\text{IAA}\;\text{in}\;1\;g\\ \;\text{of}\;\text{the}\;\text{reference}\;\text{protein})] \end{array}$$

[1].

The DIAAS of a protein reflects the availability of the most limiting IAA relative to the minimal requirement. The lowest DIAAS indicates that the corresponding IAA is the most limiting IAA of the protein, and this score is used as the DIAAS for that protein. The minimal IAA scores for a reference protein were calculated with a minimal protein requirement value of 0.66 g.kg body weight (BW).d^-1^[1] (Additional file 3). In our study, the minimal DIAAS for each IAA within the different protein-diets was calculated (Table 1). Thus, the lowest score represents the DIAAS for the corresponding protein-diet.

**Table 1:** Dietary IAA reference ratios for minimal IAA and DIAAS per protein diet

	**His**	**Ile**	**Leu**	**Lys**	**SAA**	**AAA**	**Thr**	**Val**	**Trp**	**DIAAS**
										*% (IAA)*
**5En% wheat**	1.26	1.06	1.05	0.53	1.68	1.36	1.00	0.94	1.62	53 (Lys)
**5En% wheat + 10En%:**										
**Whey + α-lac**	1.19	1.63	1.45	1.47	2.00	1.50	1.90	1.13	3.06	113 (Val)
**Soy**	1.29	1.14	1.00	0.84	1.09	1.01	1.10	0.89	1.54	84 (Lys)
**Beef**	1.78	1.38	1.23	1.44	1.65	1.79	1.55	1.12	1.33	112 (Val)
**5En% wheat + 25En%:**										
**Whey + α-lac**	1.17	1.77	1.55	1.69	2.08	1.53	2.12	1.18	3.41	117 (His)
**Soy**	1.31	1.18	1.00	0.93	0.96	1.02	1.14	0.89	1.53	89 (Val)
**Beef**	1.93	1.47	1.30	1.68	1.66	1.92	1.71	1.18	1.28	118 (Val)

α-lac, α-lactalbumin; AAA, aromatic amino acids (phenylalanine + tyrosine); DIAAS, digestible indispensable amino acid score; En%, percentage of energy; IAA, indispensable amino acid; His, histidine; Ile, isoleucine; Leu, leucine; Lys, lysine; SAA, sulphur amino acids (cysteine + methionine); Thr, threonine; Trp, tryptophan; Val, valine.

In order to assess whether subjects met their minimal IAA requirements by consuming the different protein-diets, the protein intake to meet these requirements was calculated per diet (Table 2).

**Table 2:** Calculated protein intake to meet minimal IAA requirements and measured intake over 12 days

	**Calculated**	**Measured**	**Calculated**	**Measured**
	***g.kgBW.d***^***-1***^		***g/d***	
**5En% wheat**	1.24	0.39	87.6	27.5
**15En%**				
**5En% wheat + 10En%:**				
**Whey + α-lac**	0.58	1.01	40.0	67.5
**Soy**	0.78	0.98	57.8	70.7
**Beef**	0.59	1.18	41.0	81.2
**30En%**				
**5En% wheat + 25En%:**				
**Whey + α-lac**	0.56	1.61	38.3	108.4
**Soy**	0.74	1.56	54.5	113.4
**Beef**	0.56	1.96	38.9	134.5

α-lac, α-lactalbumin; BW, body weight; En%, percentage of energy; IAA, indispensible amino acid.

$$\begin{array}{c} \text{Protein}\;\text{intake}\;\text{to}\;\text{meet}\;\text{dietary}\;\text{IAA}\;\text{requirements}\;\left(g.\text{kgBW}.d^{-1}\right)\\ \;=[\text{assumed}\;\text{dietary}\;\text{IAA}\;\text{requirement}\;(\text{minimal}:\\ \;0.66\;g.\text{kgBW}.d^{-1})/\text{DIAAS}]\text{.} \end{array}$$

### Statistical analysis

SPSS version 20 for Macintosh OS X (SPSS Inc.) was used to perform statistical analyses. Differences in subject characteristics between protein groups were assessed using Factorial ANOVA. Factorial ANOVAs with repeated measures were applied to test whether nitrogen excretion and nitrogen balance changed in response to the dietary interventions within protein groups. Differences were regarded as statistically significant if *P* < 0.05. Data are presented as mean ± SD.

Possible effects of protein source on nitrogen excretion and nitrogen balance were evaluated by calculating the differences in response elicited by the different protein diets within each protein group. Kruskal-Wallis tests were used to evaluate whether these differences were affected by source of protein. Mann-Whitney tests were used to follow-up noted differences. The Monte Carlo method was applied to estimate the significance of the Mann-Whitney tests. Effect sizes for the Mann-Whitney tests were calculated by dividing the obtained *z*-scores by the corresponding number of subjects. A Bonferroni correction was applied so all effects are reported at a 0.05 / 3 = 0.017 level of significance. Data are presented as median ± range. Differences were regarded as statistically significant if *P* < 0.05.

## Results

### Subject characteristics

Subject characteristics are summarized per protein group in Table 3. Subject characteristics did not differ between the protein groups.

**Table 3:** Subject characteristics of the whey with α-lactalbumin, soy and beef protein groups

	**Whey protein**	**Soy protein**	**Beef protein**
**Participants, M/F ( *n *)**	16/23	24/26	30/28
**Age (y)**	35 ± 17	34 ± 19	33 ± 16
**Age range (y)**	18 – 70	18 – 69	19 – 70
**Height (cm)**	172 ± 11	174 ± 10	169 ± 10
**BW (kg)**	68.6 ± 13.8	73.6 ± 12.3	69.8 ± 11.8
**BMI (kg/m**^**2**^**)**	23.1 ± 3.5	24.3 ± 3.2	24.4 ± 4.0
**BMI range (kg/m**^**2**^**)**	18.2 – 33.9	18.1 – 33.4	18.7 – 38.7
**BMR (MJ/d)**	6.5 ± 1.2	6.9 ± 1.0	6.7 ± 1.0
**PAL**	1.76 ± 0.15	1.75 ± 0.14	1.73 ± 0.16
**DER (MJ/d)**	11.4 ± 2.0	12.1 ± 2.1	11.5 ± 2.1
**TFEQ Dietary restraint score**	6 ± 4	5 ± 3	6 ± 4
**TFEQ Disinhibition score**	4 ± 2	4 ± 3	4 ± 3
**TFEQ Hunger score**	4 ± 2	4 ± 3	4 ± 3

Values shown as means ± SDs.

There were no significant differences between the protein groups (factorial ANOVA).

BMR, basal metabolic rate; BW, body weight; DER, daily energy requirement; PAL, physical activity level; TFEQ, Three-Factor Eating Questionnaire.

### Biomarker of protein intake and nitrogen balance

There was a dose-dependent increase in nitrogen excretion with increasing dietary protein content, irrespective of the protein sources (*P* = 0.001, Table 4). In each condition, no significant difference between the urine collection at days 5 and 11 were observed, which indicates stable protein intakes within the conditions over the 12-d test periods [9,10]. Nitrogen balance did not differ significantly from zero on the 5En%-protein-diet, and was positive on the 15En%- and 30En%-protein diets (*P* = 0.001, Table 4). The differences in nitrogen excretion and balance between the protein-diets were not affected by the sources of protein or by age.

**Table 4:** Nitrogen excretion and nitrogen balance per protein diet

	**Whey protein (*n*** **= 39)**	**Soy protein (*n*** **= 40)**	**Beef protein (*n*** **= 58)**
**N excretion day 11 (mg.kgBW.d**^**-1**^**)**			
**5En% (5En% wheat)**	71.2 ± 30.6^a^	70.8 ± 22.8^a^	67.9 ± 21.6^a^
**15En% (5En% wheat + 10En%)**	124.8 ± 59.0^b^	112.2 ± 47.9^b^	138.0 ± 56.8^b^
**30En% (5En% wheat + 25En%)**	203.0 ± 104.6^c^	191.0 ± 79.9^c^	217.4 ± 109.7^c^
**N balance day 11 (mg.kgBW.d**^**-1**^**)**			
**5En% (5En% wheat)**	4.2 ± 39.1^a^	-6.9 ± 28.3^a^	3.5 ± 27.6^a^
**15En% (5En% wheat + 10En%)**	47.6 ± 52.1^b^	48.5 ± 43.5^b^	50.1 ± 60.4^b^
**30En% (5En% wheat + 25En%)**	73.5 ± 69.6^c^	71.5 ± 53.9^c^	95.8 ± 110.1^c^

Values shown as means ± SDs.

Values with different superscript letters indicate significant differences between diets that differed in relative protein content, *P* < 0.05 (in the whey, in the soy, or in the beef protein diets, repeated-measures ANOVA with Bonferroni correction for pairwise post-hoc comparisons).

There were no significant differences between the protein groups (Non-parametric tests).

BW, body weight; En%, percentage of energy; N, nitrogen P, protein.

### Protein intake and DIAAS

The DIAAS for minimal IAA intake are displayed for the 5En%-protein diet, and for the 15En% and 30En%-protein diets containing whey with α-lactalbumin, soy or beef protein separately (Additional file 3). Measured protein intake from the 5En%-wheat-protein diet was lower than the intake necessary to meet the calculated minimal IAA requirements (Table 2). Measured protein intake from the 15En%- and 30En%-protein diets was sufficient to meet minimal IAA requirements, irrespective of protein source. Age did not have an effect on differences in protein intake between conditions.

In line with previous data on amino acid composition, wheat flour protein was deficient in lysine, and had a lower total IAA content than whey with α-lactalbumin, soy and beef protein (Additional file 1). A characteristic of whey and α-lactalbumin protein was the relatively high abundance of leucine and lysine. Soy protein was lower in the sulphur amino acids (SAA) cysteine and methionine. The 15En%- and 30En%-soy protein diets had a relatively lower IAA content than diets with comparable protein levels from whey with α-lactalbumin or beef sources (Additional file 2).

For the 5En%-protein diet, the lowest minimal DIAAS was obtained for lysine. Valine and histidine were the most limiting IAA of the 15En%- and 30En%-protein diets containing whey with α-lactalbumin protein (Table 1). For the 15 En%- and 30En%-protein diets containing soy, the most limiting IAA were lysine and valine respectively. The minimal DIAAS for the diets containing 15En% and 30En% from beef protein were ascribed to valine. DIAAS for minimal IAA intake were higher on the 15En%- and 30En%-protein diets compared with the 5En%-protein diet. All the added protein sources tested were relatively high quality protein sources, but the DIAAS for minimal IAA intake only exceeded 100% for the whey with α-lactalbumin and beef containing diets. In the 15En%- and 30En%-protein conditions, a higher protein intake from the soy-containing diets than from the whey with α-lactalbumin or beef containing diets was needed to meet the minimal IAA requirements.

## Discussion

This study determined whether nitrogen balance was affected and whether dietary IAA were adequately obtained from the ad libitum consumption of diets at three levels of protein from different primary sources for 12 days. The successful implementation of the dietary protein intervention on each condition in both trials was confirmed by urinary nitrogen concentrations. There was a dose-dependent increase in nitrogen excretion with increasing dietary protein content, irrespective of the protein sources. Despite protein intake with the 5En%-protein diet being below the minimal protein requirement of 0.66 g.kgBW.d^-1^[1], it was sufficient to maintain nitrogen balance over 12 days. Nitrogen balance was positive on the 15En%- and 30En%-protein diets, irrespective of dietary protein composition. However, protein intake from the 5En%-protein diet did not reach the amount necessary to meet the calculated minimal IAA requirements, but IAA were sufficiently obtained from the 15En%- and 30En%-protein diets. In the 15En%- and 30En%-protein conditions, a higher protein intake from the soy-containing diets than from the whey with α-lactalbumin or beef containing diets was needed to meet the minimal IAA requirements.

Data of this study suggest that the lower concentration of protein provided with the 5En%-protein diet was not outside the range that is feasible to maintain nitrogen balance over 12 days. The Adaptive Demands model developed by Millward may provide an explanation for this observation by proposing that the metabolic demand for amino acids comprises a fixed component and a variable adaptive component [39]. Short-term changes in protein intake are likely within the adaptive range [3]. Adaptations in protein and amino acid metabolism to changes in protein intake largely occur via changes in whole body protein turnover and amino acid oxidation [2,40]. Changes in amino acid oxidation were reflected as decreased and increased nitrogen excretion in response to the low- and high-protein diets respectively. The activity of the enzymes that regulates: 1) transamination, 2) the disposal of the carbon skeletons in intermediary metabolism, and 3) the disposal of nitrogen through the urea cycle was increased in response to high protein intake [41,42]. Nevertheless, a positive nitrogen balance was observed in the present study following the high-protein diets despite increased enzyme activities. This is in line with earlier observations [2,43-45], but does not automatically reflect an increase in protein anabolism [3]. The capacity of the body to increase amino acid anabolism through an increase in lean body mass is limited [3]. Only interventions using diets high in specific IAA, such as leucine, might be able to stimulate protein synthesis in specific target groups [46,47]. Therefore, a transient retention or loss of body nitrogen because of a labile pool of body nitrogen may contribute to adaptations in amino acid metabolism in response to changes in protein intake [48]. Transient adaptive mechanisms may be distinguished from mechanisms that maintain homeostasis in the body in the longer-term.

The calculated DIAAS for the 5En%-protein diet were well below 100%, which confirms that wheat is a low-quality protein. Especially at this low protein density, the intake of lysine was inadequate for satisfying the minimal IAA requirements. Protein intake should exceed 0.66 g.kgBW.d^-1^ to reach the minimal IAA intake. Consequently, measured intake from wheat protein being 0.39 g.kgBW.d^-1^ with the 5En%-protein diet means that minimal IAA requirements were not met. Protein intake was not spontaneously adjusted to reach the calculated optimal intake level of IAA. To meet the calculated minimal DIAAS level, subjects should have consumed ~63 MJ a day, from this diet. Interestingly, in animals, the detection of reduced concentrations of IAA in the anterior piriform cortex in the brain may result in deacetylation of the cognate transfer RNA [15]. Subsequent activation of general amino acid nonderepressing kinase 2 may phosphorylate eukaryotic initation factor 2α, a factor involved in the control of the initiation of translation in protein synthesis. This may lead to behavioral responses including under-consumption of diets that lack a minimal amount of IAA [13,16,18]. The findings of this study do not show a behavioral response in humans [9,10]. The lower concentration of protein provided in the present trials was not outside the range that is feasible to adjust protein intake. Nevertheless, a study [49] has documented acute food-choice compensation after low-protein meals in humans. After a low-protein meal, an increase in wanting and task-related signaling in the hypothalamus has been related to increased protein intake in a subsequent meal [49]. Griffioen-Roose et al. [50] showed in a 4-day study that subjects increased their protein intake in a compensatory way during ad libitum feeding after a low-protein diet (5En% from protein). Since no shift towards a higher energy intake from the 5En% protein diet compared with the 15En% protein diet was observed (9, 10), the insufficient amount of IAA clearly does not trigger a possible compensatory protein intake over 12 days. Observations from studies in developing countries show health deterioration related to low protein intake, especially of IAA, at intakes < 0.66 g.kg BW.d^-1^[51]. Thus, although we conclude that protein intake did not compensate for an insufficient IAA intake with a low-protein diet for 12 days, we do not propose that low-protein diets are sustainable.

Increasing the relative dietary protein content from whey with α-lactalbumin, soy or beef protein resulted in an improved protein quality of the diets. However, the higher DIAAS of the whey with α-lactalbumin and beef diets did not affect total daily protein intake differently compared with the lower DIAAS of the soy diets. Minimal requirements for IAA were reached with the ad libitum intake of each 15En% and 30En%-protein diet. However, a higher protein intake from the soy-containing diets than from the whey with α-lactalbumin or beef containing diets was needed to meet the minimal IAA requirements. This corresponds with the recommendations for vegetarians to consume more protein or to include a combination of different plant protein sources in the diet. Furthermore, a larger amount of protein from whey with α-lactalbumin or beef was available to fulfill roles beyond the obligatory role of maintenance. Since the subjects in this study were healthy, one of these roles may be the stimulation of muscle protein synthesis. The relative high abundance of leucine in whey with α-lactalbumin protein may be beneficial to stimulate muscle protein synthesis [46].

The DIAAS can be applied in practice to examine protein quality of food products or mixed diets. However, it should be emphasized that the calculations of the DIAAS rely on some assumptions. First, the use of digestibility coefficients based on animal data is unavoidable when human data is not available. Second, the calculated protein intake necessary to meet minimal IAA requirements is based on the assumptions of a daily minimal protein requirement. This value may vary depending on dietary protein composition and should be adjusted according to subject-specific protein requirements. Factors influencing protein and amino acid metabolism, subsequently affecting protein and IAA requirements, should be further elucidated. Longer-term intervention studies with measurements of blood concentrations of amino acids and other key factors in appetite regulation, whole body protein turnover and muscle protein synthesis would provide more insight in the changes in protein and amino acid metabolism in response to dietary protein intake. Furthermore, possible IAA sensing pathways involved in the regulation of energy and protein metabolism in humans remain to be investigated.

## Conclusion

To summarize, nitrogen balance was maintained on the 5En%-protein diet, and was positive on the 15En%- and 30En%-protein diets over 12 days. Despite this, the protein intake from the 5En%-protein diet did not reach the amount necessary to meet the calculated minimal IAA requirements, but IAA were sufficiently obtained from the 15En%- and 30En%-protein diets. In the 15En%- and 30En%-protein conditions, a higher protein intake from the soy-containing diets than from the whey with α-lactalbumin or beef containing diets was needed to meet the minimal IAA requirements. In conclusion, protein intake did not compensate for an insufficient indispensable amino acid intake with a low-protein diet for 12 days.

## Abbreviations

BW: Body weight; DIAAS: Digestible indispensable amino acid score; En%: Percentage of energy; IAA: Indispensable amino acids; VAS: Visual analog scale.

## Competing interests

All of the authors declare that they have no competing interest.

## Authors’ contributions

The study was designed by MSWP, RDM, EAM, and SYT. EAM analyzed the data, performed the statistical analyses, and wrote the paper. MSWP, RDM, and SYT contributed to the interpretation of the data and reviewed the manuscript. MSWP had primary responsibility for final content. All authors read and approved the final manuscript.

## Supplementary Material

Additional file 1 IAA composition per type of protein. Click here for file

Additional file 2 True ileal digestible IAA and total IAA content per protein diet. Click here for file

Additional file 3 IAA reference pattern for minimal requirements. Click here for file

## References

[B1] FAO Dietary Protein Quality Evaluation in Human Nutrition: Report of an FAO Expert Consultation FAO Food and Nutrition paper 92 2013 Rome: FAO 26369006

[B2] Tome D Bos C Dietary protein and nitrogen utilization J Nutr 2000 130 1868S 1873S 10867065 10.1093/jn/130.7.1868S

[B3] Millward DJ Identifying recommended dietary allowances for protein and amino acids: a critique of the 2007 WHO/FAO/UNU report Br J Nutr 2012 108 Suppl 2 S3 S21 23107542 10.1017/S0007114512002450

[B4] Millward DJ Amino acid scoring patterns for protein quality assessment Br J Nutr 2012 108 Suppl 2 S31 S43 23107544 10.1017/S0007114512002462

[B5] Tome D Criteria and markers for protein quality assessment - a review Br J Nutr 2012 108 Suppl 2 S222 S229 23107532 10.1017/S0007114512002565

[B6] Elango R Ball RO Pencharz PB Recent advances in determining protein and amino acid requirements in humans Br J Nutr 2012 108 Suppl 2 S22 S30 23107531 10.1017/S0007114512002504

[B7] Millward DJ Macronutrient intakes as determinants of dietary protein and amino acid adequacy J Nutr 2004 134 1588S 1596S 15173435 10.1093/jn/134.6.1588S

[B8] Gosby AK Conigrave AD Lau NS Iglesias MA Hall RM Jebb SA Brand-Miller J Caterson ID Raubenheimer D Simpson SJ Testing protein leverage in lean humans: a randomised controlled experimental study PLoS One 2011 6 e25929 22022472 10.1371/journal.pone.0025929 PMC3192127

[B9] Martens EA Lemmens SG Westerterp-Plantenga MS Protein leverage affects energy intake of high-protein diets in humans Am J Clin Nutr 2013 97 86 93 23221572 10.3945/ajcn.112.046540

[B10] Martens EA Tan SY Dunlop MV Mattes RD Westerterp-Plantenga MS Protein leverage effects of beef protein on energy intake in humans Am J Clin Nutr 2014 99 1397 1406 24760974 10.3945/ajcn.113.078774

[B11] Aparecida de Franca S Dos Santos MP Garofalo MA Navegantes LC Kettelhut Ido C Lopes CF Kawashita NH Low protein diet changes the energetic balance and sympathetic activity in brown adipose tissue of growing rats Nutrition 2009 25 1186 1192 19535223 10.1016/j.nut.2009.03.011

[B12] Du FY Higginbotham DA White BD Food intake, energy balance and serum leptin concentrations in rats fed low-protein diets J Nutr 2000 130 514 521 10702578 10.1093/jn/130.3.514

[B13] Gietzen DW Hao S Anthony TG Mechanisms of food intake repression in indispensable amino acid deficiency Annu Rev Nutr 2007 27 63 78 17328672 10.1146/annurev.nutr.27.061406.093726

[B14] Gietzen DW Rogers QR Nutritional homeostasis and indispensable amino acid sensing: a new solution to an old puzzle Trends Neurosci 2006 29 91 99 16406138 10.1016/j.tins.2005.12.007

[B15] Hao S Sharp JW Ross-Inta CM McDaniel BJ Anthony TG Wek RC Cavener DR McGrath BC Rudell JB Koehnle TJ Gietzen DW Uncharged tRNA and sensing of amino acid deficiency in mammalian piriform cortex Science 2005 307 1776 1778 15774759 10.1126/science.1104882

[B16] Maurin AC Jousse C Averous J Parry L Bruhat A Cherasse Y Zeng H Zhang Y Harding HP Ron D Fafournoux P The GCN2 kinase biases feeding behavior to maintain amino acid homeostasis in omnivores Cell Metab 2005 1 273 277 16054071 10.1016/j.cmet.2005.03.004

[B17] Meyer JH Interactions of dietary fiber and protein on food intake and body composition of growing rats Am J Physiol 1958 193 488 494 13533580 10.1152/ajplegacy.1958.193.3.488

[B18] Ross CM Sharp JW Gietzen DW eIF2 alpha related signaling of amino acid deficiency in brain areas Faseb J 2004 18 A145

[B19] White BD Porter MH Martin PJ Protein selection, food intake, and body composition in response to the amount of dietary protein Physiology & Behavior 2000 69 383 389 10913775 10.1016/s0031-9384(99)00232-2

[B20] Harris JA Benedict FG A biometric study of human basal metabolism Proc Natl Acad Sci U S A 1918 4 370 373 16576330 10.1073/pnas.4.12.370 PMC1091498

[B21] Baecke JA Burema J Frijters JE A short questionnaire for the measurement of habitual physical activity in epidemiological studies Am J Clin Nutr 1982 36 936 942 7137077 10.1093/ajcn/36.5.936

[B22] Johansson G Westerterp KR Assessment of the physical activity level with two questions: validation with doubly labeled water Int J Obes (Lond) 2008 32 1031 1033 18392036 10.1038/ijo.2008.42

[B23] Edholm OG Energy balance in man studies carried out by the Division of Human Physiology, National Institute for Medical Research J Hum Nutr 1977 31 413 431 591718

[B24] Vicennati V Ceroni L Gagliardi L Gambineri A Pasquali R Comment: response of the hypothalamic-pituitary-adrenocortical axis to high-protein/fat and high-carbohydrate meals in women with different obesity phenotypes J Clin Endocrinol Metab 2002 87 3984 3988 12161547 10.1210/jcem.87.8.8718

[B25] Sorensen LB Moller P Flint A Martens M Raben A Effect of sensory perception of foods on appetite and food intake: a review of studies on humans Int J Obes Relat Metab Disord 2003 27 1152 1166 14513063 10.1038/sj.ijo.0802391

[B26] Westerterp KR Perception, passive overfeeding and energy metabolism Physiol Behav 2006 89 62 65 16516250 10.1016/j.physbeh.2005.12.014

[B27] Westerterp-Plantenga MS Fat intake and energy-balance effects Physiol Behav 2004 83 579 585 15621063 10.1016/j.physbeh.2004.07.027

[B28] Westerterp KR Verboeket-van de Venne WP Westerterp-Plantenga MS Velthuis-te Wierik EJ de Graaf C Weststrate JA Dietary fat and body fat: an intervention study Int J Obes Relat Metab Disord 1996 20 1022 1026 8923159

[B29] FAO Amino-Acid Content of Foods and Biological Data on Proteins. FAO Food and Nutrition series 1970 Rome: FAO 5537267

[B30] United States Department of Agriculture Agricultural Research Service. SR25 Page Reports. Version current September 2013 Available from: http://www.ars.usda.gov/Services/docs.htm?docid=22770 (cited June 2014)

[B31] Rutherfurd SM Bains K Moughan PJ Available lysine and digestible amino acid contents of proteinaceous foods of India Br J Nutr 2012 108 Suppl 2 S59 S68 23107549 10.1017/S0007114512002280

[B32] Deglaire A Moughan PJ Animal models for determining amino acid digestibility in humans - a review Br J Nutr 2012 108 Suppl 2 S273 S281 23107538 10.1017/S0007114512002346

[B33] Moughan PJ Gilani S Rutherfurd SM Tomé D True ileal Amino Acid Digestibility Coefficients for Application in the Calculation of Digestible Indispensable Amino Acid Score (DIAAS) in Human Nutrition 2012 Rome: FAO

[B34] Rowan AM Moughan PJ Wilson MN Maher K Tasman-Jones C Comparison of the ileal and faecal digestibility of dietary amino acids in adult humans and evaluation of the pig as a model animal for digestion studies in man Br J Nutr 1994 71 29 42 8312239 10.1079/bjn19940108

[B35] FAO/WHO Protein Quality Evaluation: Report of the Joint FAO/WHO Expert Consultation. FAO Food and Nutrition paper 51 1991 Rome: FAO

[B36] Moughan PJ Butts CA Rowan AM Deglaire A Dietary peptides increase endogenous amino acid losses from the gut in adults Am J Clin Nutr 2005 81 1359 1365 15941887 10.1093/ajcn/81.6.1359

[B37] Wolfe RR The role of dietary protein in optimizing muscle mass, function and health outcomes in older individuals Br J Nutr 2012 108 Suppl 2 S88 S93 23107552 10.1017/S0007114512002590

[B38] Wolfe RR Perspective: optimal protein intake in the elderly J Am Med Dir Assoc 2013 14 65 66 23128023 10.1016/j.jamda.2012.09.017

[B39] Millward DJ An adaptive metabolic demand model for protein and amino acid requirements Br J Nutr 2003 90 249 260 12908885 10.1079/bjn2003924

[B40] Deutz NE Wolfe RR Is there a maximal anabolic response to protein intake with a meal? Clin Nutr 2013 32 309 313 23260197 10.1016/j.clnu.2012.11.018 PMC3595342

[B41] Harper AE Miller RH Block KP Branched-chain amino acid metabolism Annu Rev Nutr 1984 4 409 454 6380539 10.1146/annurev.nu.04.070184.002205

[B42] Harper AE Some recent developments in the study of amino acid metabolism Proc Nutr Soc 1983 42 437 449 6361772 10.1079/pns19830051

[B43] Garlick PJ McNurlan MA Patlak CS Adaptation of protein metabolism in relation to limits to high dietary protein intake Eur J Clin Nutr 1999 53 Suppl 1 S34 S43 10365979 10.1038/sj.ejcn.1600742

[B44] Price GM Halliday D Pacy PJ Quevedo MR Millward DJ Nitrogen homeostasis in man: influence of protein intake on the amplitude of diurnal cycling of body nitrogen Clin Sci (Lond) 1994 86 91 102 8306557 10.1042/cs0860091

[B45] Pannemans DL Halliday D Westerterp KR Kester AD Effect of variable protein intake on whole-body protein turnover in young men and women Am J Clin Nutr 1995 61 69 74 7825541 10.1093/ajcn/61.1.69

[B46] van Loon LJ Leucine as a pharmaconutrient in health and disease Curr Opin Clin Nutr Metab Care 2012 15 71 77 22037013 10.1097/MCO.0b013e32834d617a

[B47] Millward DJ Knowledge gained from studies of leucine consumption in animals and humans J Nutr 2012 142 2212S 2219S 23077184 10.3945/jn.111.157370

[B48] Munro HN General Aspects of the Regulation of Protein Metabolism by Hormones 1964 New York: Academic press

[B49] Born JM Martens MJ Lemmens SG Goebel R Westerterp-Plantenga MS Protein v. carbohydrate intake differentially affects liking- and wanting-related brain signalling Br J Nutr 2013 109 376 381 22643242 10.1017/S0007114512001092

[B50] Griffioen-Roose S Mars M Siebelink E Finlayson G Tome D de Graaf C Protein status elicits compensatory changes in food intake and food preferences Am J Clin Nutr 2012 95 32 38 22158729 10.3945/ajcn.111.020503 PMC3238463

[B51] WHO/FAO/UNU Protein and Amino Acid Requirements in Human Nutrition. Report of a Joint WHO/FAO/UNU Expert Consultation WHO Technical Report Series No 935 2007 Geneva: WHO 18330140

