# Supplementary material for: No protein intake compensation for insufficient indispensable amino acid intake with a low-protein diet for 12 days
Source: Nutr Metab (Lond). 2014 Aug 20;11:38. doi: 10.1186/1743-7075-11-38 (PMC4147096; doi:10.1186/1743-7075-11-38)
Supplement: Additional file 1 — IAA composition per type of protein. [file 1743-7075-11-38-S1.doc]

| **Additional file 1 IAA composition per type of protein** | | | | | | | | | |
| --- | --- | --- | --- | --- | --- | --- | --- | --- | --- |
| ***mg/g protein*** | | | | | | | | | |
|  | **His** | **Ile** | **Leu** | **Lys** | **SAA** | **AAA** | **Thr** | **Val** | **Trp** |
| **Wheat flour** | 20 | 34 | 66 | 27 | 39 | 55 | 26 | 41 | 11 |
| **Whey** | 16 | 59 | 94 | 84 | 43 | 51 | 63 | 51 | 16 |
| **α-lac** | 27 | 57 | 107 | 103 | 60 | 81 | 48 | 43 | 38 |
| **Soy** | 20 | 37 | 60 | 46 | 18 | 68 | 28 | 35 | 10 |
| **Beef** | 34 | 48 | 81 | 89 | 40 | 80 | 46 | 50 | 8 |
| **α-lac, α-lactalbumin; AAA, aromatic amino acids (phenylalanine + tyrosine); His, histidine; IAA, indispensable amino acid; Ile, isoleucine; Leu, leucine; Lys, lysine; SAA, suphur amino acids (cysteine + methionine); Thr, threonine; Trp, tryptophan; Val, valine.** | | | | | | | | | |
