# Supplementary material for: No protein intake compensation for insufficient indispensable amino acid intake with a low-protein diet for 12 days
Source: Nutr Metab (Lond). 2014 Aug 20;11:38. doi: 10.1186/1743-7075-11-38 (PMC4147096; doi:10.1186/1743-7075-11-38)
Supplement: Additional file 2 — True ileal digestible IAA and total IAA content per protein diet. [file 1743-7075-11-38-S2.doc]

| **Additional file 2 True ileal digestible IAA and total IAA content per protein diet** | | | | | | | | | | |
| --- | --- | --- | --- | --- | --- | --- | --- | --- | --- | --- |
| ***mg/g protein*** | | | | | | | | | | |
|  | **His** | **Ile** | **Leu** | **Lys** | **SAA** | **AAA** | **Thr** | **Val** | **Trp** | **Total** |
| **5En% wheat** | 19 | 32 | 62 | 24 | 37 | 52 | 23 | 37 | 10 | 294 |
|  |  |  |  |  |  |  |  |  |  |  |
| **5En% wheat + 10En%:** |  |  |  |  |  |  |  |  |  |  |
| **Whey + α-lac** | 18 | 49 | 85 | 66 | 44 | 57 | 44 | 44 | 18 | 425 |
| **Soy** | 19 | 34 | 59 | 38 | 24 | 61 | 25 | 35 | 9 | 305 |
| **Beef** | 27 | 41 | 73 | 65 | 36 | 68 | 36 | 44 | 8 | 397 |
|  |  |  |  |  |  |  |  |  |  |  |
| **5En% wheat + 25 En%:** |  |  |  |  |  |  |  |  |  |  |
| **Whey + α-lac** | 18 | 53 | 91 | 76 | 46 | 58 | 49 | 46 | 20 | 457 |
| **Soy** | 20 | 35 | 59 | 42 | 21 | 64 | 26 | 35 | 9 | 311 |
| **Beef** | 29 | 44 | 76 | 76 | 37 | 73 | 39 | 46 | 8 | 428 |
| **α-lac, α-lactalbumin; AAA, aromatic amino acids (phenylalanine + tyrosine); En%, percentage of energy; His, histidine; IAA, indispensable amino acid; Ile, isoleucine; Leu, leucine; Lys, lysine; SAA, sulphur amino acids (cysteine + methionine); Thr, threonine; Trp, tryptophan; Val, valine.** | | | | | | | | | | |
