# Supplementary material for: No protein intake compensation for insufficient indispensable amino acid intake with a low-protein diet for 12 days
Source: Nutr Metab (Lond). 2014 Aug 20;11:38. doi: 10.1186/1743-7075-11-38 (PMC4147096; doi:10.1186/1743-7075-11-38)
Supplement: Additional file 3 — IAA reference pattern for minimal requirements. [file 1743-7075-11-38-S3.doc]

| **Additional file 3 IAA reference pattern for minimal requirements** | | | | | | | | |
| --- | --- | --- | --- | --- | --- | --- | --- | --- |
| ***mg/g protein*** | | | | | | | | |
| **His** | **Ile** | **Leu** | **Lys** | **SAA** | **AAA** | **Thr** | **Val** | **Trp** |
| 15 | 30 | 59 | 45 | 22 | 38 | 23 | 39 | 6 |
| **AAA, aromatic amino acids (phenylalanine + tyrosine); His, histidine; IAA, indispensable amino acid; Ile, isoleucine; Leu, leucine; Lys, lysine; SAA, sulphur amino acids (cysteine + methionine); Thr, threonine; Trp, tryptophan; Val, valine.** | | | | | | | | |
